# Miniaturized Dual and Quad Port MIMO Antenna Variants Featuring Elevated Diversity Performance for UWB and 5G-Midband Applications

**DOI:** 10.3390/mi16060716

**Published:** 2025-06-17

**Authors:** Karthikeyan Ramanathan, Srivatsun Gopalakrishnan, Thrisha Chandrakanthan

**Affiliations:** 1 Department of ECE, Kumaraguru College of Technology, Coimbatore 641049, India; thrisha.21ec@kct.ac.in; 2 Department of ECE, PSG College of Technology, Coimbatore 641001, India; gsn.ece@psgtech.ac.in

**Keywords:** MIMO antenna, 2-port, 4-port, 5G mid-band, UWB, ECC, TARC, MEG

## Abstract

The growing demand for high-speed and high-capacity wireless communication has intensified the need for compact, wideband, and efficient MIMO antenna systems, particularly for 5G mid-band and UWB applications. This article presents a miniaturized dual and quad port MIMO antenna design optimized for 5G mid-band (n77/n78/n79/n96/n102) and Ultra-Wideband (UWB) applications without employing any decoupling structures between the radiating elements. The 2-port configuration features two closely spaced symmetric monopole elements (spacing < λ_max_/2), promoting efficient use of space without degrading performance. An FR4 substrate (εr = 4.4) is used for fabrication with a compact size of 30 × 41 × 1.6 mm^3^. This layout is extended orthogonally and symmetrically to form a compact quad-port variant with dimensions of 60 × 41 × 1.6 mm^3^. Both designs offer a broad operational bandwidth from 2.6 GHz to 10.8 GHz (8.2 GHz), retaining return loss (S_XX_) below −10 dB and strong isolation (S_XY_ < −20 dB at high frequencies, <−15 dB at low frequencies). The proposed MIMO antennas demonstrate strong performance and excellent diversity characteristics. The two-port antenna achieves an average envelope correlation coefficient (ECC) of 0.00204, diversity gain (DG) of 9.98 dB, and a mean effective gain difference (MEG_ij_) of 0.3 dB, with a total active reflection coefficient (TARC) below −10 dB and signal delay variation under 0.25 ns, ensuring minimal pulse distortion. Similarly, the four-port design reports an average ECC of 0.01432, DG of 9.65 dB, MEG_ij_ difference below 0.3 dB, and TARC below −10 dB, confirming robust diversity and MIMO performance across both configurations.

## 1. Introduction

Today’s wireless communication systems are pivotal in enabling high-throughput applications including WLANs (Wireless Local Area Networks), HDTV (High-Definition Television), WBANs (Wireless Body Area Networks), and PANs (Personal Area Networks). Ultra-Wideband (UWB) technology is a strong contender for short-range wireless communication, offering the potential for rapid data transfer [1]. The popularity of this technology grew significantly after the Federal Communications Commission (FCC) released its first report in 2002. While UWB technology enables rapid transmission in indoor environments, it faces challenges such as multipath fading, interference, and constrained spectrum availability [2]. The rapid advancement of wireless communication systems emphasizes not only achieving higher data rates but also ensuring strong error performance [3]. Achieving both high data rates and low error rates simultaneously is challenging due to multipath propagation and fading effects. As a result, a balance must be struck between bandwidth efficiency and power efficiency. While channel modeling can help mitigate signal fading, it often leads to increased complexity in transceiver hardware [4]. An alternative approach to balance transceiver hardware complexity and signal fading is through antenna design innovations [5]. Foschini et al. first introduced the concept of UWB-MIMO antennas, which have gained popularity in the past decade for next-generation wireless systems [6]. Designing ultrawideband antennas that function within 3.1–10.6 GHz presents significant challenges due to wide bandwidth requirements [7,8]. The time-domain performance must remain stable across the entire range, with factors like dispersion and group delay becoming important alongside frequency-domain characteristics [9,10]. The development of UWB-MIMO antennas also faces challenges such as size, cost, and integration, while the miniaturization of antennas often leads to increased mutual coupling and reduced isolation [11,12]. Achieving high isolation, robust diversity performance, and ultra-wideband characteristics simultaneously remains a core challenge in UWB-MIMO antenna design [13,14,15]. To tackle these challenges, extensive research has been done on dual-port and quad-port MIMO variants employing various decoupling mechanisms and structural modifications. Bahmanzadeh et al. (2021) introduced a 2-port antenna using a stub of ‘T’ shape to enhance isolation [16]. Babu and Anuradha (2021) introduced a miniaturized MIMO variant employing a ground perturbation to suppress inter-element coupling [17]. Chen et al. (2019) combined circularly polarized dielectric resonator elements with an electromagnetic bandgap (EBG) surface to achieve excellent diversity performance for 5G applications [18]. Du et al. (2024) further improved isolation by leveraging hybrid polarization in their MIMO patch antenna using a polarization conversion parasitic structure [19]. In the 4-port domain, Thongbor et al. (2016) demonstrated a compact 4-port antenna incorporating a common ground and arrow-shaped slot etching aided isolation enhancement [20]. Liu et al. (2020) integrated neutralization lines into their triple-band MIMO antenna design, achieving good isolation and diversity gain [21]. Malviya and Chouhan (2019) presented a 4-port antenna using a shared radiator along with stepped ground for enhanced diversity effects, advancing miniaturization and performance for WLAN applications [22]. Mohanty and Sahu (2021) introduced a fractal-inspired 4-port UWB MIMO antenna integrating coupling resonators as port-isolators to reduce mutual coupling while maintaining Wi-Max rejection and wideband performance [23].

While these designs have achieved significant improvements, they often rely on additional decoupling structures such as EBG, stubs, or neutralization lines, parasitic elements, Split Ring Resonators (SRR), and Inder Digital Capacitors (IDC) which can complicate fabrication, increase design complexity, and sometimes limit further miniaturization. This underlines a crucial need for antenna designs that can deliver wide bandwidth, low envelope correlation coefficient (ECC), high diversity performance, and strong isolation without relying on any decoupling structures between the radiating elements.

In response, this study introduces novel miniaturized dual port and quad port MIMO antenna variants that achieve enhanced diversity performance across the 5G mid-band and UWB spectrum without including decoupling structures in between the radiators. The antenna designs leverage intrinsic structural symmetry, orthogonal mode excitation, and careful spatial arrangement of the elements to naturally suppress mutual coupling while maintaining compactness and simplicity. The proposed antennas demonstrate wide impedance bandwidth, low ECC, high diversity gain, and stable time-domain performance, offering a streamlined solution well-suited for next-generation wireless communication systems.

## 2. Dual-Port MIMO Antenna

### 2.1. Geometry and Design Evolution

**Figure 1:**
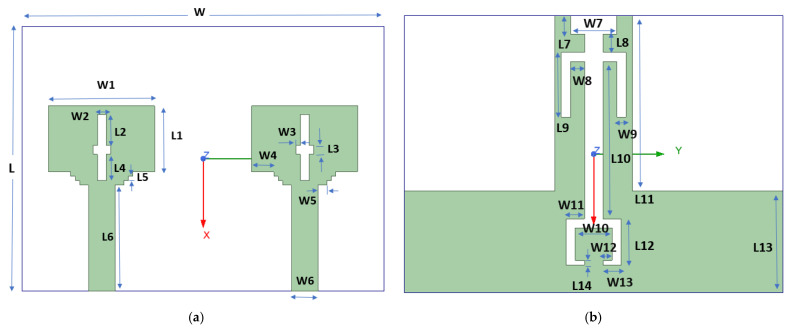
Dual-port geometry (**a**) top layout (**b**) ground layout.

This section presents the comprehensive geometry and design progression of the presented dual-port MIMO variant. Figure 1a displays the top view and Figure 1b displays the ground view with dimensions 41 × 30 × 1.6 mm^3^. Table 1 provides a detailed breakdown of the antenna’s dimensions.

**Figure 2:**
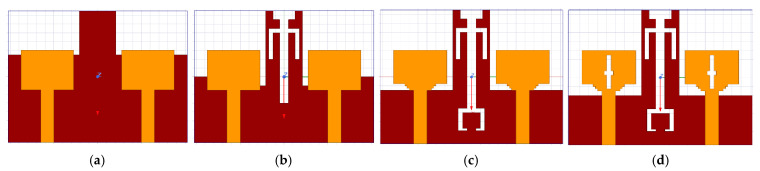
(**a**–**d**) Stage-wise advancement in the proposed antenna configuration.

**Figure 3:**
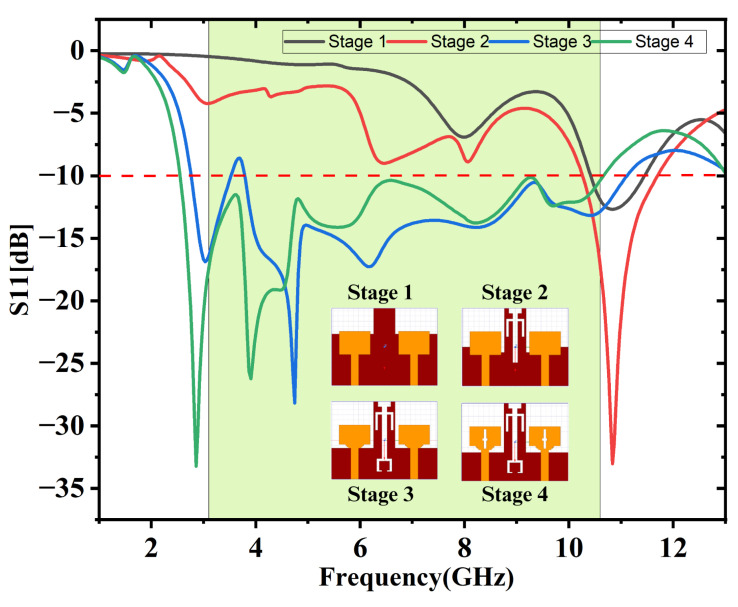
Simulated S11 vs. frequency for four-stage antenna evolution.

This finalized antenna design was achieved through a four-stage development process with parametric analysis. The sequential evolution of the antenna across these four stages is illustrated in Figure 2a–d. The antenna elements are excited by 50 Ω microstrip lines. Stage 1 involves the creation of a basic rectangular radiating element accompanied by a slightly modified ground which is illustrated in Figure 2a. Computed impedance profile is illustrated in Figure 3, revealing that the 10 dB bandwidth achieved fails to encompass the full UWB range spanning 3.1 GHz to 10.6 GHz. At this stage, the electric field is mainly confined near the feed point and the central area of the radiating patch with minimal spreading of surface currents resulting in narrow bandwidth. In Stage 2, a U-shaped slot oriented up-side down and a linear I-shaped slot are embedded into the ground, as illustrated in Figure 2b. This variation leads to a notable improvement in the impedance bandwidth, as evidenced in the simulation results displayed in Figure 3. The current flow on the ground plane was disturbed by the introduction of slots, creating new resonance paths, enabling a broader distribution of the electric field, and helping to enhance the antenna’s bandwidth. However, the desired full UWB range is still not achieved. In Stage 3, two small rectangular notches resembling staircase-like cuts are added to the bottom corners of the radiating patch along with a slot of C-shape which is carved into the antenna’s ground. The updated impedance characteristics are shown in Figure 3 indicating further enhancement in bandwidth. These alterations improve impedance matching by altering current paths and enabling more efficient E-field radiation near the patch edges and the ground slot, though the complete UWB range remains uncovered. Finally in Stage 4, two additional rectangular slots are introduced at the center of the antenna elements, one oriented vertically and the other horizontally so that they intersect to form a cross-shaped slot.

**Figure 4:**
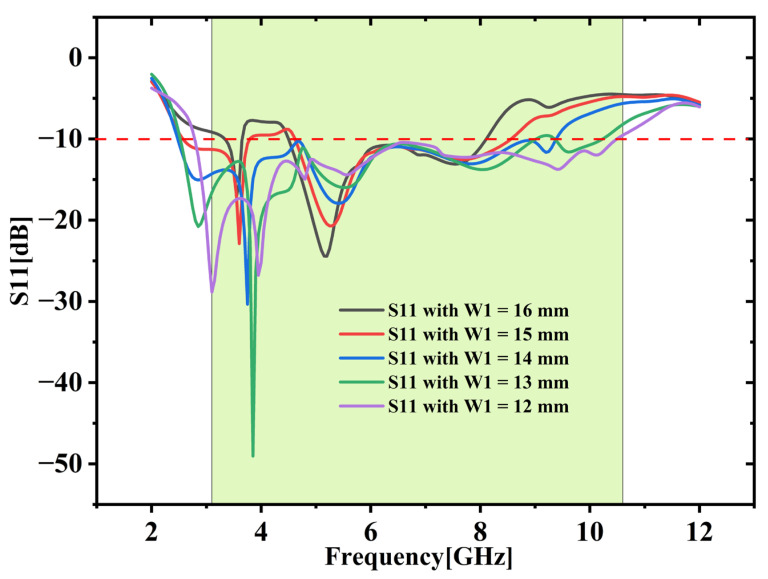
Parametric analysis (W1 = 12 mm to 16 mm).

**Figure 5:**
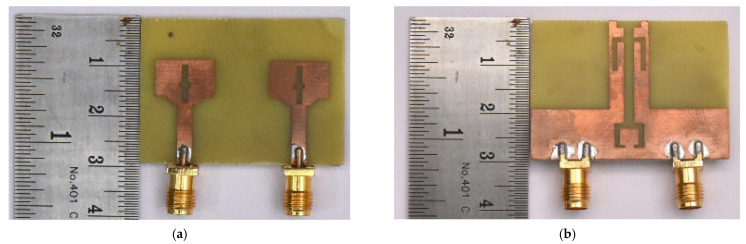
Fabricated antenna (**a**) top view (**b**) ground view.

This structure significantly alters the electric-field distribution by enabling stronger coupling between modes and introducing multi-resonant behavior and helps to flatten the reflection coefficient curve and extend the bandwidth. Using the stage 4 structure, a parametric study was conducted by altering the length W1 varied between 10 mm and 14 mm with 1 mm intervals to examine its effect on impedance matching and resonant frequencies. The comparative results for the different values of W1 are illustrated in Figure 4. The analysis reveals that adjusting the W1 length leads to a noticeable increase in bandwidth and a reduction in reflection. Notably, when W1 = 12 mm, the design achieves the widest bandwidth along with the lowest reflection coefficient. This final modification combined with parametric analysis allows the antenna design to effectively cover the full Ultra-Wideband (UWB) spectrum. Figure 5 shows the fabricated prototype equipped with 50-ohm SMA connectors.

### 2.2. Antenna Measurements

**Figure 6:**
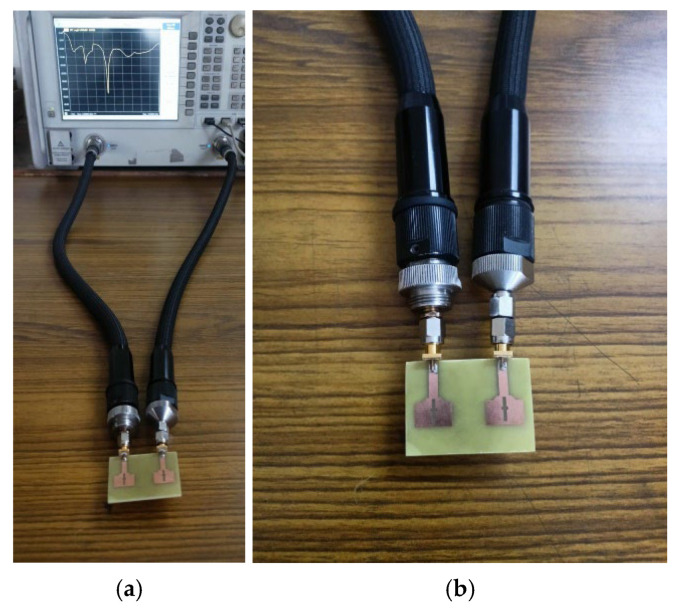
(**a**) Impedance characteristics measurement setup with Agilent N5247A: A.09.90.02 VNA and (**b**) closer view of fabricated antenna with VNA connectors.

**Figure 7:**
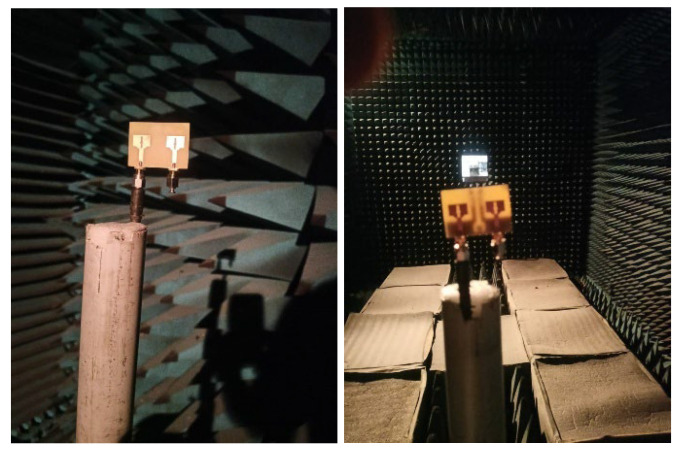
Radiation characteristics measurement setup using anechoic chamber.

The testing setup employed for measuring the impedance and radiation performance is illustrated in Figure 6 and Figure 7. The impedance characteristics (S_XX_ and S_XY;_ X and Y values are from 1 to 2) are measured using Agilent N5247A: A.09.90.02 Vector network Analyzer (VNA) illustrated in Figure 6. To measure S_XX_ (reflection) parameter, port X of the fabricated antenna is linked to port 1 of the VNA for signal excitation, and other ports are connected with 50-ohm terminations. For S_XY_ (transmission) parameter measurement ports X and Y of the fabricated antenna are connected to ports 1 and 2 of the VNA.

### 2.3. Validation of S-Parameters: Simulation vs. Measurement (S_XX_ and S_XY_)

**Figure 8:**
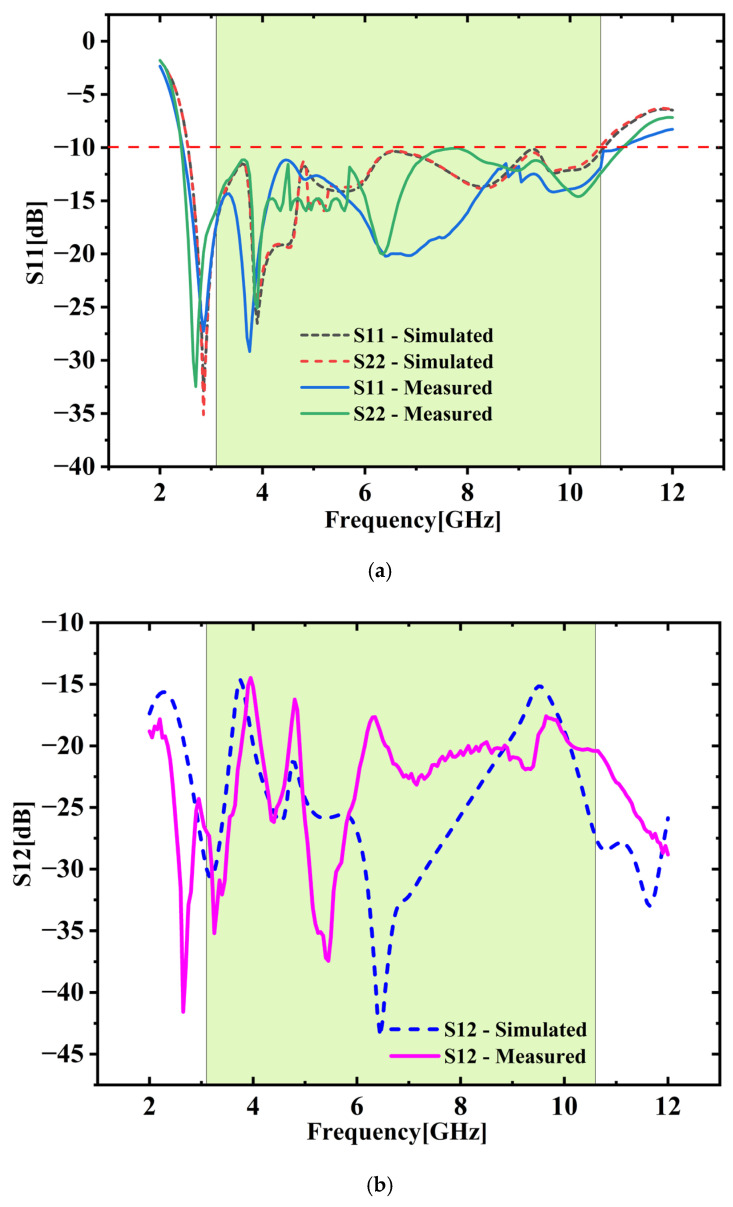
(**a**) S_XX_ vs. frequency (**b**) S_XY_ vs. frequency.

Figure 8a illustrates the relation between the measured and simulated S_XX_ parameters. This plot provides a comparative visualization of simulated and experimentally measured S-parameters, allowing for an assessment of their agreement or deviation. The measured S_XX_ values closely match the simulated data, demonstrating fair agreement. The developed 2-port MIMO antenna supports a broad operating bandwidth spanning 2.6 GHz to 10.8 GHz, based on the S_XX_ < −10 dB threshold. Figure 8b displays the comparison of transmission coefficients (S_XY_). The data show that S_XY_ remains below −20 dB at higher frequencies and under −15 dB at lower frequencies, confirming that the 2-port MIMO antenna maintains excellent isolation characteristics. Minor variations in the measured results in both cases may arise from factors such as challenges associated with SMA connectors, manufacturing precision limits, and the relatively narrow ground plane set against the connected cable may affect the current flow pattern [24].

### 2.4. Group Delay

**Figure 9:**
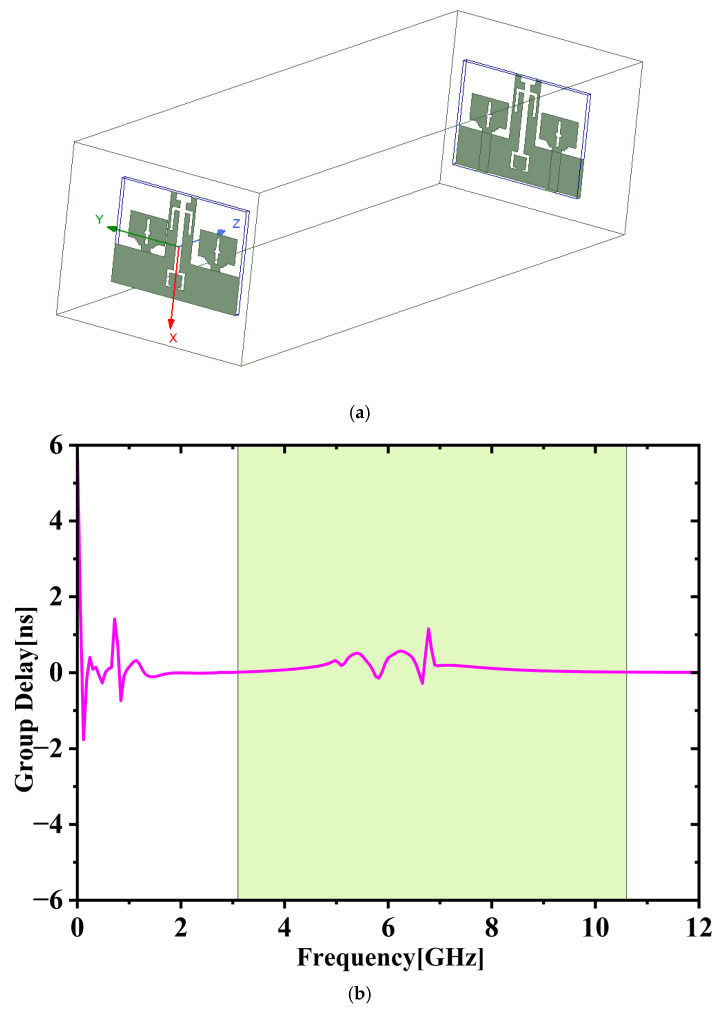
(**a**) Antenna arrangement for group delay measurement. (**b**) Group delay vs. frequency.

Group delay serves as a key parameter in evaluating the performance of wideband antennas, as it reflects the extent to which a signal waveform is altered during transmission. Figure 9a,b depict the experimental setup for the transmit-receive antenna arrangement and group delay performance. To guarantee far-field measurement conditions, the receiving antenna is positioned 50 cm away, with the far-field distance determined using Equation (1), where ‘B’ represents the separation between the transmit and receive antennas, and ‘S’ denotes the largest dimension of the presented antenna. Figure 9b illustrates the group delay remains uniform throughout the full UWB spectrum.

(1) $$B=\frac{2S^{2}}{\lambda }$$

The delay variation stays within 0.25 ns, indicating that the transmitted signal experiences very little distortion across the operating range.

### 2.5. Diversity Parameters

#### 2.5.1. Envelope Correlation Coefficient (ECC)

ECC quantifies the level of correlation between signals and indicates possible performance degradation caused by mutual coupling. Equation (2) calculates ECC using scattering (S) parameters between different antenna ports [25,26]. A smaller ECC value signifies improved isolation and superior diversity performance.

(2) $$\mathrm{ECC}=\frac{\left|S_{ii}^{*}S_{ij}+S_{ji}^{*}S_{jj}\right|}{\left(1-{{|S}_{ii}|}^{2}-{{|S}_{ji}|}^{2}\right)(1-{{|S}_{jj}|}^{2}-{{|S}_{ij}|}^{2})}$$

**Figure 10:**
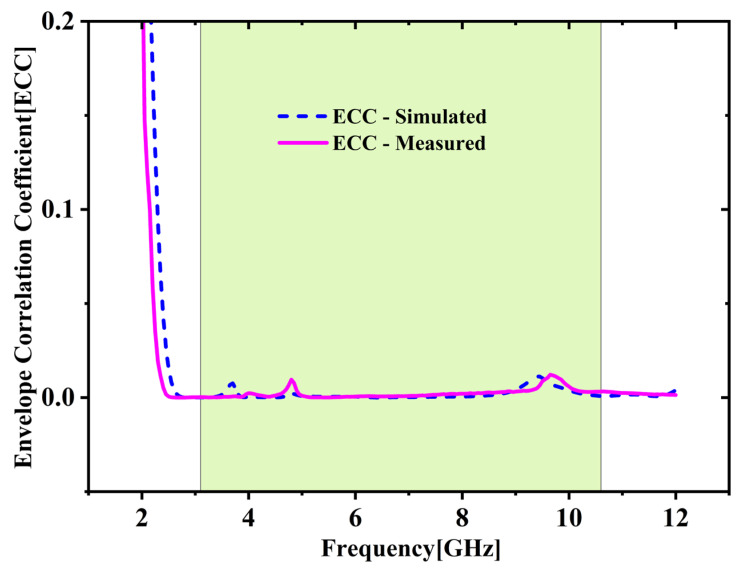
Comparison of simulated and measured ECC.

Figure 10 compares simulated and measured ECC. It shows ECC remains under 0.01 throughout the intended 3.1 to 10.6 GHz band. The total average ECC across all ports is calculated to be 0.002045, which is notably less than the widely accepted threshold of 0.5, indicating excellent diversity performance [25,26].

#### 2.5.2. Diversity Gain (DG)

**Figure 11:**
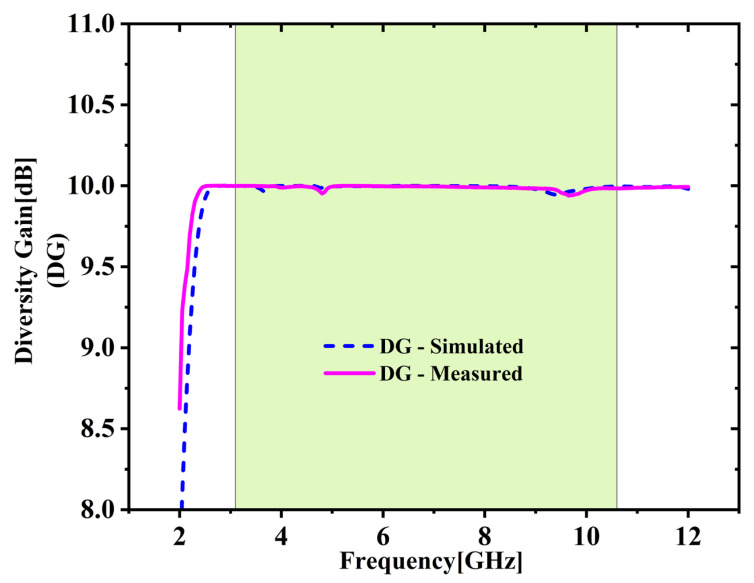
Diversity gain (DG) comparison.

It measures the enhancement in reception achieved through antenna diversity. It is calculated using Equation (3) [25,26], which relies on the characteristics of ECC. Figure 11 demonstrates that both the simulated and measured DG values remain consistently near the ideal benchmark of 10 dB throughout the full UWB spectrum. In this case, the lowest observed DG is 9.93 dB, and the highest is 9.99 dB, resulting in an average DG of 9.98 dB. Hence the system effectively improves signal quality by utilizing multiple antennas.

(3) $$\mathrm{DG}=10\sqrt{1-{\left(ECC\right)}^{2}}$$

Low ECC values along with high diversity gain demonstrate strong isolation between the antenna elements. This outcome is the result of spatial separation and a well-optimized ground plane structure. These design features limit electromagnetic interaction and suppress surface current coupling.

#### 2.5.3. Total Active Reflection Coefficient (TARC)

**Figure 12:**
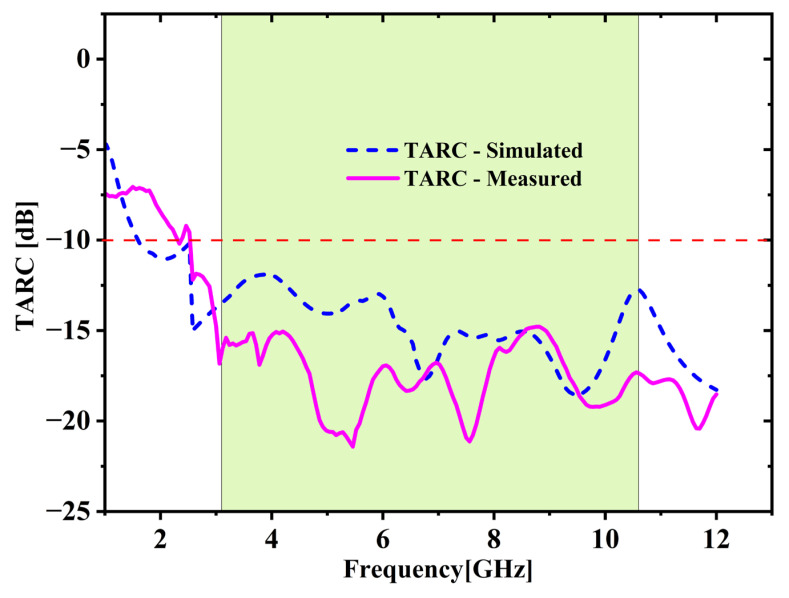
Total active reflection coefficient (TARC) comparison.

It evaluates the complete power reflected when all ports are excited simultaneously with identical amplitude and phase. For the developed 2-port MIMO configuration, TARC is determined through Equation (4) [25,26]. Figure 12 illustrates the TARC outcomes obtained from both simulation and measurement. The TARC remains under −10 dB within UWB spectrum for both cases, indicating superior diversity performance of the developed antenna.

(4) $$\mathrm{TARC}=\sqrt{\frac{1}{N}\sum_{i=1}^{N}{|\sum_{j=1}^{N}S_{ij}|}^{2}}$$

#### 2.5.4. Mean Effective Gain (MEG)

**Figure 13:**
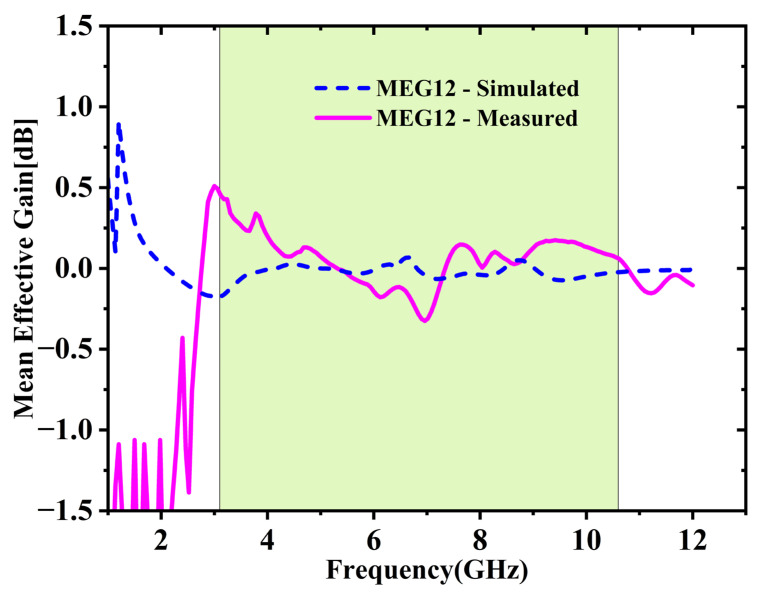
Simulated vs. measured MEG comparison.

An important parameter for assessing efficiency of individual elements within a MIMO system, especially in practical multipath conditions. It is calculated using Equation (5) [27]. For consistent and balanced diversity performance, the MEG difference between antenna elements should remain within 3 dB [27]. The difference computed with Equation (6) [27] shown in Figure 13, remains under 0.3 dB throughout the UWB frequency range, indicating that the antenna operates consistently and is well balanced.

(5) $${MEG}_{i}=0.5[1-\sum_{j=1}^{N}{{|S}_{ij}|}^{2}]$$

(6) $${MEG}_{ij}={MEG}_{i}-{MEG}_{j}$$

### 2.6. Radiation Pattern

**Figure 14:**
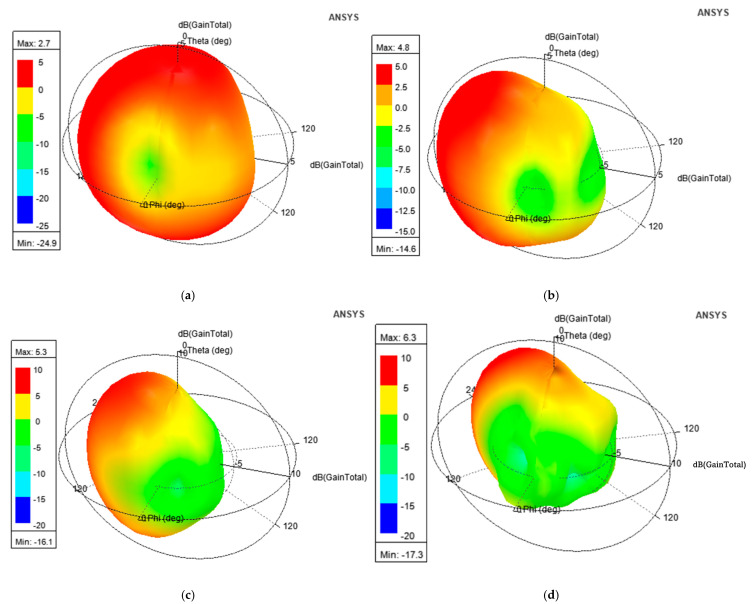
Three-dimensional (3D) radiation patterns at (**a**) 4.0 GHz; (**b**) 6.125 GHz; (**c**) 9.185 GHz; (**d**) 10.5 GHz.

Figure 14a–d display the 3D radiation patterns pertaining to 4, 6.125, 9.185, and 10.5 GHz. The plots indicate nearly omnidirectional radiation with the gain ranging from 2.7 dB to a peak of 6.3 dB.

A comprehensive comparison between the reported designs and the presented UWB MIMO antenna with emphasis on diversity metrics, bandwidth, and gain is presented in Table 2. Additionally, the computed diversity parameters of the presented UWB antenna are summarized in Table 3.

## 3. Quad-Port MIMO Antenna

### 3.1. Antenna Geometry and Design Evolution

**Figure 15:**
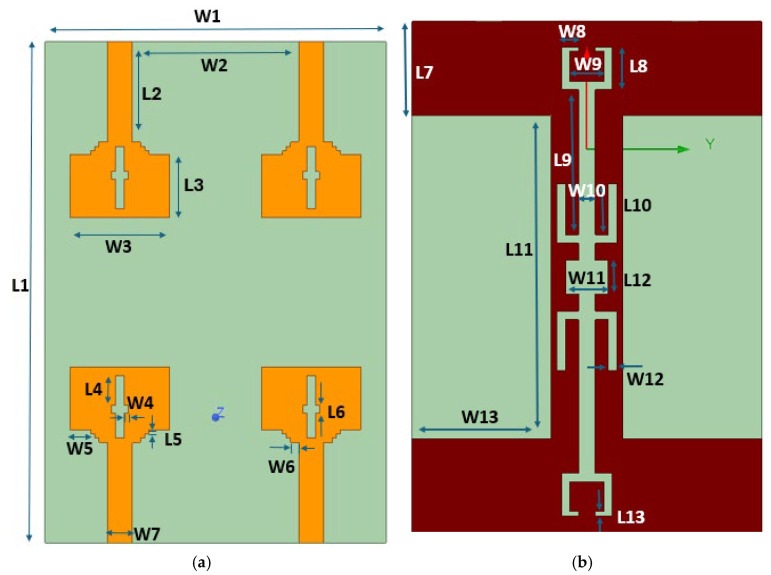
Quad-port geometry (**a**) top layout (**b**) ground layout.

**Figure 16:**
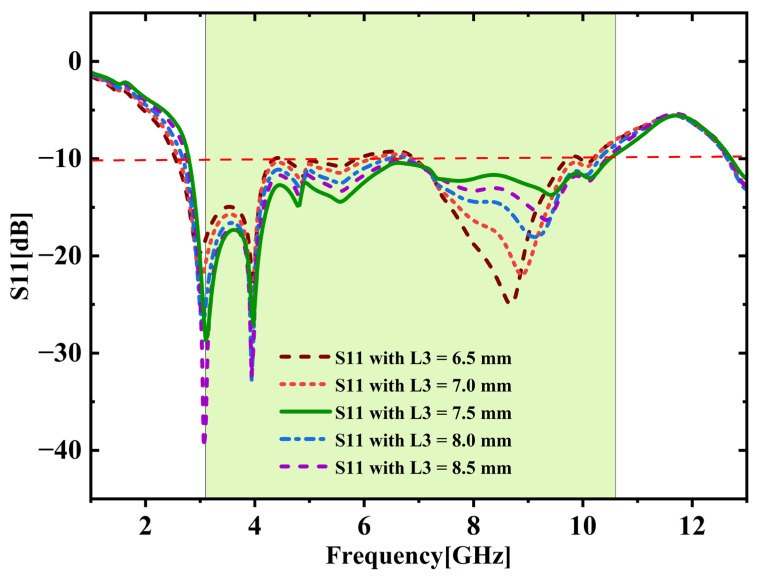
Parametric analysis (L3 = 6.5 mm to 8.5 mm).

**Figure 17:**
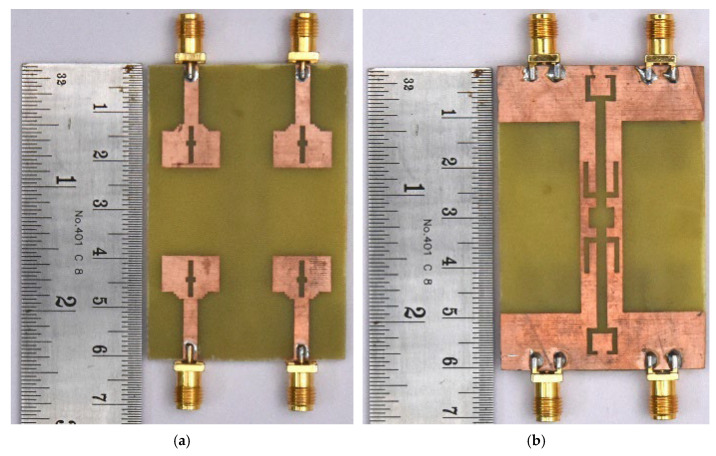
Fabricated prototype (**a**) top view and (**b**) ground view.

This section elaborates a structured and comprehensive analysis of the design methodology and performance assessment of the presented quad-port MIMO variant. The optimized dual-element configuration in the previous section is extended orthogonally in a symmetrical manner to realize a compact 4—element MIMO structure. Figure 15 shows the complete structure. The final dimensions are 60 × 41 × 1.6 mm^3^. A 50-ohm feedline is used to power each element. A parametric study was performed by varying the parameter ‘L3’ between 6.5 mm and 8.5 mm with increments of 0.5 mm. The results are illustrated in Figure 16. Among those tested values, L3 = 7.5 mm yielded the broadest bandwidth of 8.2 (2.6 GHz to 10.8 GHz). The finalized design dimensions are populated in Table 4. Figure 17 depicts the fabricated 4-port MIMO antenna prototypes equipped with 50-ohm SMA connectors.

### 3.2. Antenna Measurements

**Figure 18:**
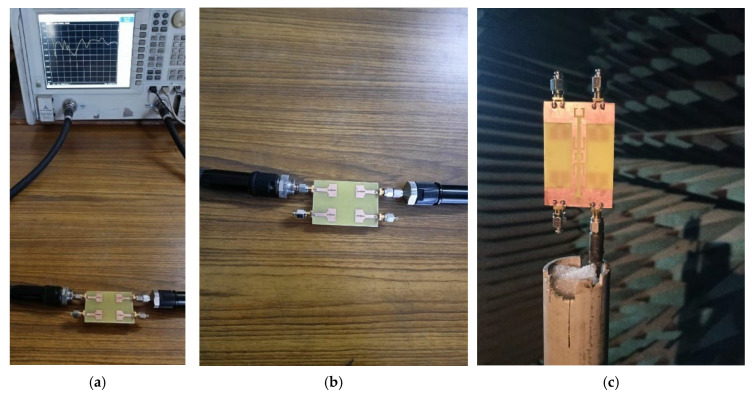
(**a**) Measurement setup for Impedance characteristics. (**b**) Closer view of fabricated antenna with VNA connectors. (**c**) Radiation characteristics measurement setup using anechoic chamber.

The testing setup employed to evaluate the impedance and radiation characteristics is shown in Figure 18a–c. The impedance parameters (S_XX_ and S_XY_, where X and Y range from 1 to 4) are recorded using Vector Network Analyzer, as depicted in Figure 18. For S_XX_ (reflection) measurements, port X of the fabricated antenna is connected with the first port VNA while the others are connected with 50-ohm terminations. For S_XY_ (transmission) measurements, the antenna’s X and Y ports are connected with ports 1 and 2 of the VNA, respectively.

### 3.3. Validation of S-Parameters: Simulation vs. Measurement (S_XX_ and S_XY_)

**Figure 19:**
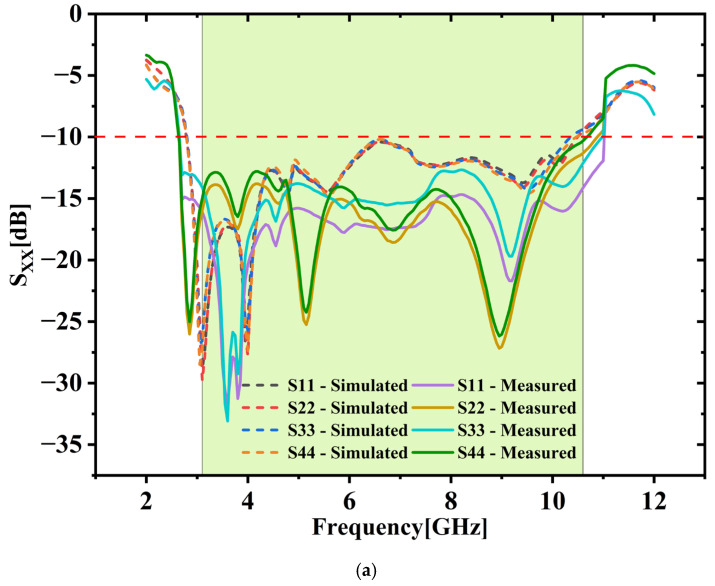

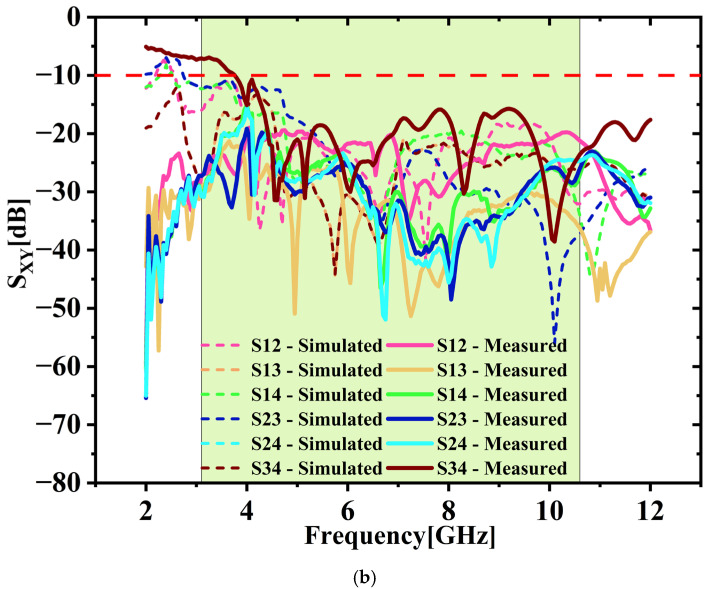
(**a**) S_XX_ vs. frequency (**b**) S_XY_ vs. frequency.

Figure 19a,b compare S_XX_ and S_XY_ parameters both simulated and measured. Measured results closely match the simulations, demonstrating strong agreement. The fabricated antenna clearly achieves a bandwidth of 8.2 GHz (with SXX < −10 dB) along with excellent isolation (SXY < −15 dB) throughout UWB range.

### 3.4. Diversity Analysis: ECC, DG, TARC and MEG

**Figure 20:**
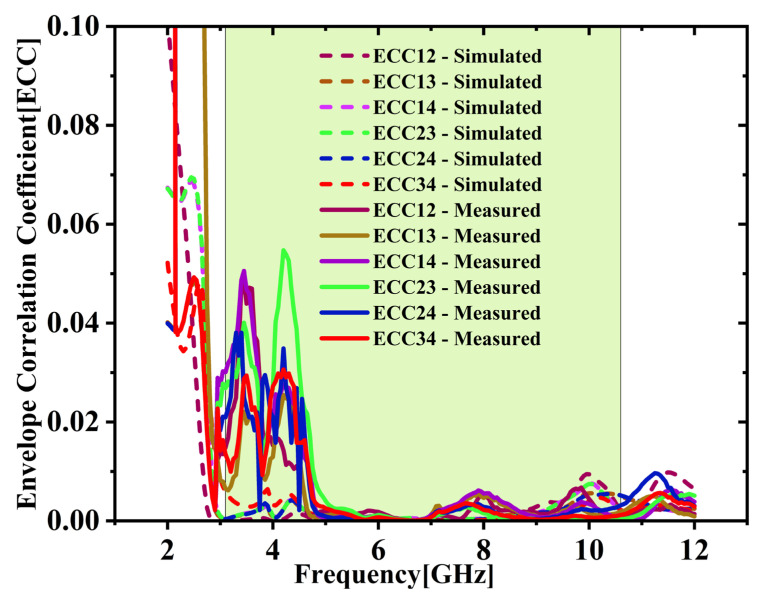
Simulated vs. measured ECC comparison.

**Figure 21:**
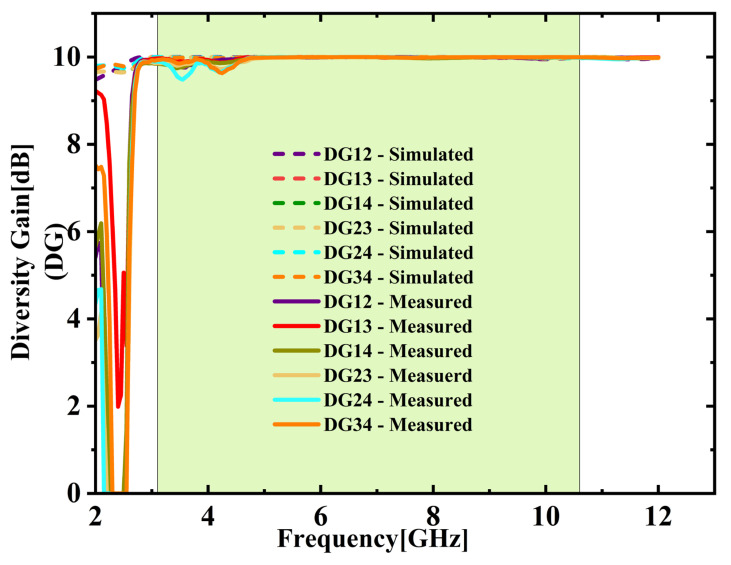
Directive gain (DG) comparison.

**Figure 22:**
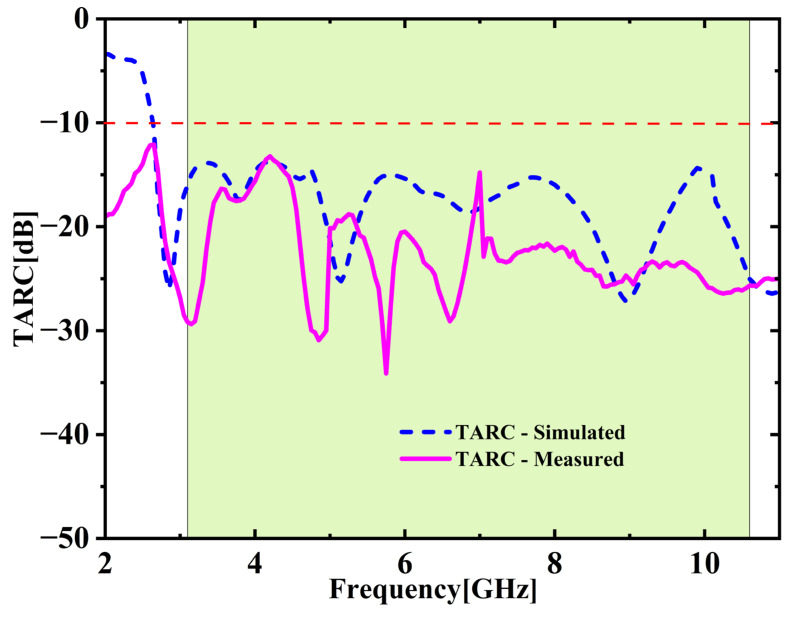
Total active reflection coefficient (TARC) comparison.

**Figure 23:**
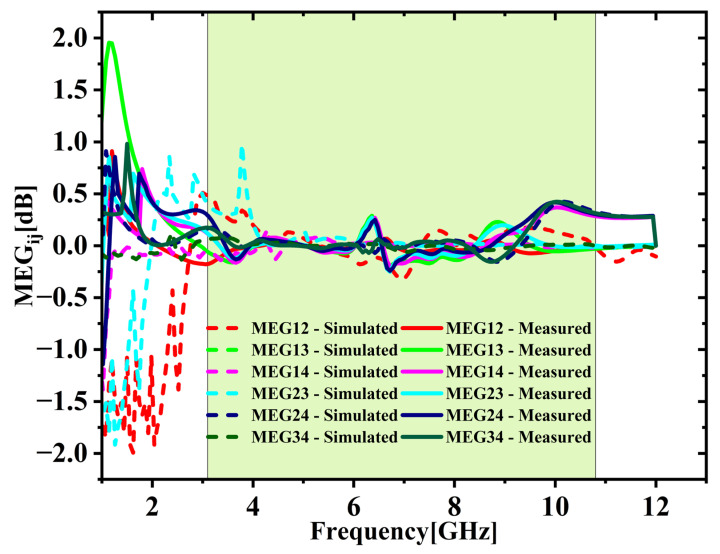
Simulated vs. measured MEG comparison.

The ECC between various port combinations is derived with the S-parameters utilizing Equation (2) [25,26]. As depicted in Figure 20, both simulation and experimental results indicate that ECC remains under 0.05 throughout the UWB spectrum. Average ECC is 0.01432, which is far below the 0.5 limit, indicating excellent diversity. Figure 21 shows the DG values stay close to the optimal 10 dB across the full UWB band. The DG ranges from 9.31 to 9.99 dB with an average of 9.65 dB across the desired UWB band. This confirms effective signal enhancement using multiple antenna elements. TARC is evaluated based on Equation (4) [25,26]. Figure 22 presents both simulated and measured outcomes, each indicating a TARC is under −10 dB. This validates the antenna’s effective diversity performance. The MEG and MEG difference between the antenna elements of the proposed design are computed with the help of Equations (5) and (6) [27]. For balanced diversity, the MEG difference should be within 3 dB. As shown in Figure 23, the calculated difference stays below 0.3 dB across the UWB band. This confirms the antenna’s stable and well-balanced performance.

### 3.5. Radiation Pattern

**Figure 24:**
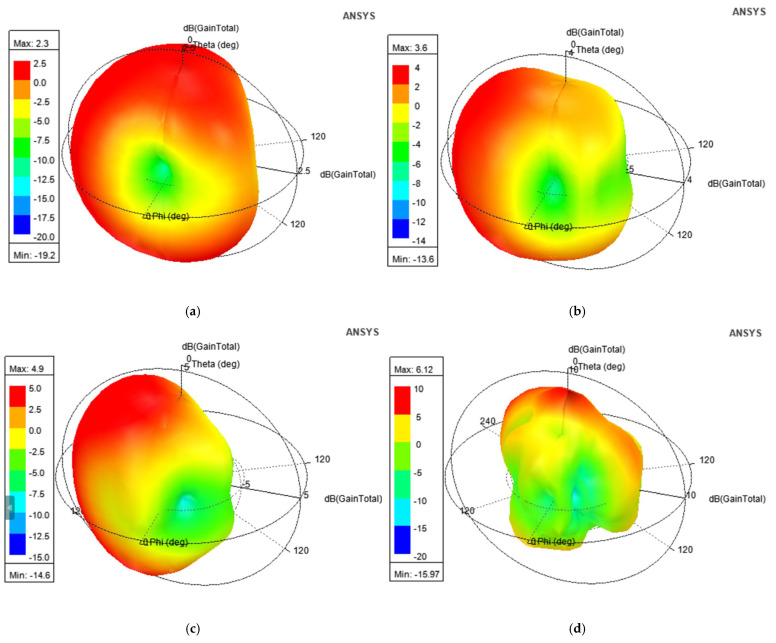
Three-dimensional (3D) radiation patterns at (**a**) 4.0 GHz; (**b**) 6.125 GHz; (**c**) 9.185 GHz; (**d**) 10.5 GHz.

Figure 24a–d display the 3D radiation patterns pertaining to 4, 6.125, 9.185, and 10.5 GHz. The plots indicate nearly omnidirectional radiation with a gain ranging from 2.3 dB to a peak of 6.12 dB.

A comprehensive comparison between the existing designs and the presented antenna with emphasis on diversity metrics, bandwidth, and gain is presented in Table 5. Additionally, the computed diversity parameters of the presented UWB antenna across different frequencies are listed in Table 6.

## 4. Conclusions

Compact dual-port and quad-port MIMO antennas have been developed to enhance overall efficiency of 5G Midband (n77/n78/n79/n96/n102) and UWB communication systems, offering improved diversity and better isolation. Both designs provide a broad impedance bandwidth of 8.2 GHz, spanning 2.6 GHz to 10.8 GHz, with reflection coefficient (S_XX_) consistently below –10 dB across the band. Both configurations demonstrate excellent strong inter-element isolation, where coupling (Sxy) remains under –15 dB at lower bands and drops beneath –20 dB at elevated frequencies. The MIMO antennas, both two-port and four-port, show excellent performance with low ECC, high Diversity Gain, and minimal MEG difference. TARC remains below the threshold, and signal delay variation is kept under 0.25 ns. These metrics ensure stable and efficient MIMO operation. Overall, the proposed antennas are compact and efficient solutions for 5G mid-band (C-band) and UWB MIMO systems. Future work will scale UWB-MIMO to larger arrays (4 × 4, 8 × 8) using advanced isolation methods (metamaterials, EBG, neutralization lines) and flexible, reconfigurable substrates for wearable and adaptive systems.

## Figures and Tables

**Table 1:** Proposed 2-port antenna dimension details.

Parameters	Measurement (mm)	Parameters	Measurement (mm)	Parameters	Measurement (mm)
L	30	L10	17	W5	1
L1	7.5	L11	19	W6	3
L2	3.5	L12	5	W7	5
L3	1	L13	11	W8	1.5
L4	3	L14	0.5	W9	1
L5	0.5	W	41	W10	4
L6	12	W1	12	W11	12
L7	2	W2	1	W12	1
L8	2	W3	0.5	W13	2
L9	7	W4	2.5		

**Table 2:** Comparison of antenna characteristics: proposed design vs. existing literature.

Ref No/Published Year	Size (mm^3^)	UWB Bandwidth (GHz)	Isolation (dB)	ECC	DG (dB)	TARC (dB)	MEG (dB)	Peak Gain (dBi)	No. of Ports
[28]/2023	47 × 38 × 0.8	2.9–11.0	20	0.003	9.99	<0	≈1	4.1	2
[29]/2022	30 × 18 × 1.6	4.3–15.63	20	<0.007	9.96	NR *	NR	5.3	2
[30]/2020	38.5 × 38.5 × 1.6	3.0–11.0	25	0.047	9.8	NR	NR	6.0	2
[31]/2019	59 × 55 × 8.1	3.0–7.0	18	<0.21	9.64	NR	NR	4	2
[32]/2018	50 × 30 × 1.6	3.0–16.0	16	<0.01	NR	<0	≈1	3.0	2
[33]/2009	50 × 100 × 1.6	2.0–6.0	15	<0.01	9.6	NR	≈1	6.0	2
P.A. ^1^	30 × 41 × 1.6	2.6–10.8	20	<0.007	9.96	<−10	0.3	6.3	2

* NR—Not Reported; ^1^ P.A.—Proposed Antenna.

**Table 3:** Assessment of performance metrics at various frequencies based on S-parameter analysis.

Diversity Parameters	Selected Frequencies (GHz)					
	4	5	7	8	9	10
ECC	0.00234	0.00096	0.0008	0.00203	0.00318	0.00582
TARC [dB]	−11.957	−14.048	−16.337	−15.537	−16.449	−16.462
MEG [dB]	0.1872	0.04518	−0.3100	0.0050	0.1260	0.13069
DG [dB]	9.98827	9.99519	9.99558	9.98983	9.98411	9.97085

**Table 4:** Proposed antenna dimension details.

Parameters	Measurement (mm)	Parameters	Measurement (mm)	Parameters	Measurement (mm)
L1	60	L10	6	W6	1
L2	12	L11	38	W7	3
L3	7.5	L12	4	W8	2
L4	3	L13	0.5	W9	4
L5	0.5	W1	41	W10	2
L6	1	W2	40	W11	5
L7	11	W3	12	W12	1
L8	5	W4	0.5	W13	16
L9	17	W5	2.5		

**Table 5:** Comparison of antenna characteristics: proposed design vs. existing literature.

Ref No/Published Year	Size (mm^3^)	UWB Bandwidth (GHz)	Isolation (dB)	ECC	DG (dB)	TARC (dB)	MEG (dB)	Gain (dBi)	No. of Ports
[34]/2024	40 × 40 × 1.6	2.51–18	16	<0.1	≈10	NR *	NR	3.5	4
[35]/2022	60 × 60 × 1.6	3.0–11.0	20	<0.02	9.98	<−10	NR	3.4	4
[36]/2022	45 × 45 × 1.6	3.1–13.1	17	<0.02	9.9985	<−25	NR	4	4
[37]/2020	80 × 80 × 1.6	2.1–20	25	<0.02	9.99	NR	NR	5.8	4
[38]/2019	58 × 58 × 1.6	3.0–13.5	22	<0.008	≈10	NR	NR	2.9	4
[39]/2018	75.19 × 75.19 × 1.6	3.1–17.3	13	<0.1	NR	NR	NR	5.5	4
[40]/2014	60 × 60 × 1.6	2.7–10.6	15	<0.063	NR	NR	NR	3.5	4
P.A. ^1^	60 × 41 × 1.6	2.6–10.8	15	<0.05	9.65	<−10	0.3	6.12	4

* NR—Not Reported ^1^ P.A.—Proposed Antenna.

**Table 6:** Assessment of performance metrics at various frequencies based on S-parameter analysis.

Diversity Parameters	Selected Frequencies (GHz)					
	4	5	7	8	9	10
ECC12	0.00002	0.00074	0.00017	0.00006	0.00286	0.00942
TARC [dB]	−14.697	−21.360	−18.238	−15.949	−27.040	−14.773
MEG12 [dB]	0.1872	0.0451	−0.3100	0.0276	0.1260	0.1306
DG12 [dB]	9.9998	9.99626	9.9991	9.9996	9.9857	9.9528

## Data Availability

All original findings discussed in this study are contained within the article and any additional questions can be addressed to the corresponding author.
